# The Role of Glial Cells in Neurobiology and Prion Neuropathology

**DOI:** 10.3390/cells13100832

**Published:** 2024-05-14

**Authors:** Arielle Hay, Katriana Popichak, Julie Moreno, Mark Zabel

**Affiliations:** 1 Division of Intramural Research, Laboratory of Persistent Viral Diseases, Rocky Mountain Laboratories, National Institute of Allergy and Infectious Diseases, National Institutes of Health, Hamilton, MT 59840, USA; 2 Prion Research Center, Department of Microbiology, Immunology and Pathology, College of Veterinary Medicine and Biomedical Sciences, Colorado State University, Fort Collins, CO 80523, USA; katriana.popichak@colostate.edu (K.P.); julie.moreno@colostate.edu (J.M.); mark.zabel@colostate.edu (M.Z.); 3 Department of Environmental and Radiological Health Sciences, College of Veterinary Medicine and Biomedical Sciences, Colorado State University, Fort Collins, CO 80523, USA

**Keywords:** prions, astrocytes, microglia, neurodegeneration, neuropathology

## Abstract

Prion diseases are rare and neurodegenerative diseases that are characterized by the misfolding and infectious spread of the prion protein in the brain, causing progressive and irreversible neuronal loss and associated clinical and behavioral manifestations in humans and animals, ultimately leading to death. The brain has a complex network of neurons and glial cells whose crosstalk is critical for function and homeostasis. Although it is established that prion infection of neurons is necessary for clinical disease to occur, debate remains in the field as to the role played by glial cells, namely astrocytes and microglia, and whether these cells are beneficial to the host or further accelerate disease. Here, we review the current literature assessing the complex morphologies of astrocytes and microglia, and the crosstalk between these two cell types, in the prion-infected brain.

## 1. What Are Prions?

Prion diseases may just be the black sheep of infectious diseases. Unlike bacterial or viral infection, the disease agent arises from the host itself in the form of the cellular prion protein (PrP^C^). PrP^C^ can be coerced to misfold from an alpha-helical form to a form rich in beta-sheets, which acts as a catalyst to induce the misfolding and aggregation of more host PrP^C^ [1]. The misfolded, infectious form of the protein is denoted as PrP^Sc^. The “protein only hypothesis” states that PrP^Sc^ is able to bind PrP^C^ and induce conformational changes, leading to an autocatalytic reaction that leads to an exponential production of PrP^Sc^ [2]. The initial misfolding can be sporadic, due to a genetic mutation in the protein, or due to acquired infection by PrP^Sc^ [3]. PrP^C^ is expressed ubiquitously in tissue, but it has the highest expression in the brain, particularly in astrocytes and neurons [4]. PrP^Sc^ aggregation disrupts homeostasis in these cells and leads to fatal neurodegeneration. Prion diseases are also called transmissible spongiform encephalopathies (TSEs), as the main hallmark of disease, apart from abundant protein aggregation in the brain, is the sponge-like appearance of brain tissue that is caused by vacuolization and referred to as spongiform change [5].

PrP is expressed in all mammalian cells as a glycoprotein that is anchored to the surface of the cell via a glycosylphosphatidylinositol (GPI) anchor [6]. The secondary structure of PrP is conserved between mammalian species [3]. PrP^Sc^ replicates itself and forms oligomers, which can lead to fibrillization. These fibrils continue to lengthen and clump together to form amyloid plaques. The intra- and extracellular deposits of these macromolecular structures are the defining characteristics of prion disease [2].

The role of oligomers, fibrils, and amyloid in prion pathogenesis remains controversial [7,8]. Fibril formation may actually be a mechanism that evolved to protect against the toxic effects of oligomerization [9]. Prion oligomerization, fibrillization, and aggregation occur before the disease onset. By the time the disease can be clinically recognized, brain regions rich in PrP^Sc^ are already undergoing neurodegeneration [2].

Being a relatively new field (the term “prion” was first coined by Stanley Prusiner in a paper published in Science in 1982) [10], there is still much uncharted territory when it comes to how the central nervous system responds to prion infection, as well as what pathways to target to best inhibit disease progression. To this day, prion diseases remain invariably fatal.

## 2. Cells of the Brain

Neurodegenerative diseases are characterized by the degeneration of neurons. Neurons are critical for sending signals throughout the central nervous system to regulate autonomic behaviors, motor movement, and cognition. Neurons make up almost half of the cells within the brain [11], and neurodegeneration leads to losses of memory, motor functions, and eventually paralysis and death. Although critical, neurons cannot function without the involvement of three other key cell types in the brain: oligodendrocytes, astrocytes, and microglia. Oligodendrocytes compose the myelin sheath that wraps around a neuron’s axon, allowing for the rapid transmission of action potentials through a process called saltatory conduction. Loss of myelin is associated with neuronal dysfunction, such as in multiple sclerosis, but there is little evidence that oligodendrocytes can be infected with PrP^Sc^ or have any significant involvement in prion disease [12]. They have been largely ignored in the field of prion disease.

**Figure 1:**
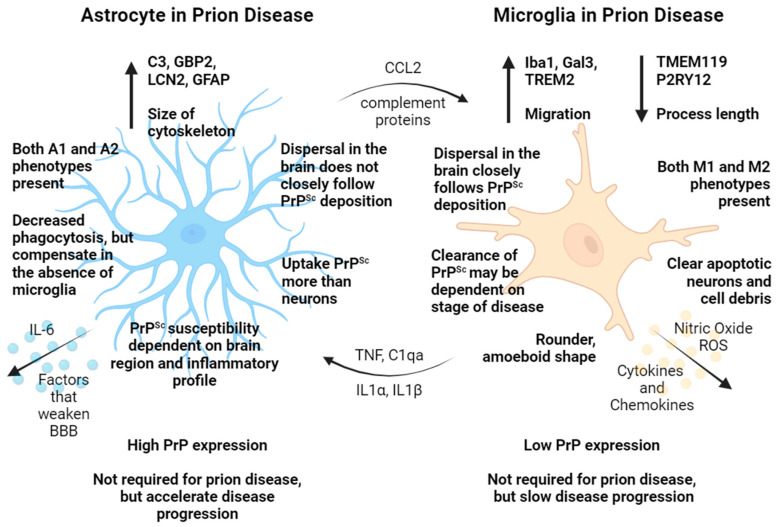
Overview of the roles of astrocytes and microglia in prion pathogenesis.

Astrocytes are also principal regulators of neuronal function, and they play a large role in prion pathogenesis (Figure 1). These cells regulate communications between neurons and the brain’s blood vessels and maintain the blood–brain barrier, which is composed of vascular endothelial cells, tight junctions, basement membranes, smooth muscle cells, and astrocytes themselves [13]. Astrocytes are critical in maintaining the homeostasis of neurons by regulating potassium and glutamate levels and neurotransmitters [14,15]. Astrocytes also regulate the survival and function of neurons and can induce the pruning of neuronal synapses by increasing complement component 1q (C1q) production in synapses. This binds to the complement component 3a (C3a) receptor on microglia, which prune the synapses via the classical compliment cascade. This can be triggered by the activation of astrocytes and increased production of astrocytic C3, which can induce more phagocytosis of neuronal synapses by microglia. Further neuronal damage can initiate a positive feedback loop and, when left unchecked, leads to neuronal loss [13,16].

The phenotypes of glial cells fluctuate depending on the environment of the brain. Injury and inflammation in the brain can induce astrocytes to shift from a homeostatic phenotype to a “reactive” phenotype. This is characterized by an increase in the size of their cytoskeleton, increased expression of glial fibrillary acidic protein (GFAP), upregulation of many genes, particularly those involved in inflammatory pathways, extension of their processes, and formation of glial scars. When astrocytes become reactive, they release factors that increase the permeability of the blood–brain barrier and allow immune cells from the periphery to infiltrate [13,17,18].

Astrocytes demonstrate a few morphological and phenotypical changes in response to insult and injury in the absence of microglia. Classic studies have demonstrated that the factors secreted by microglia, such as interleukin 1α (IL1α) and tumor necrosis factor alpha (TNFα), induce astrocyte reactivity, and in the absence of microglia, astrocytes remain relatively unresponsive to environmental changes and do not reach full states of activation [16,19]. Once triggered by microglia, astrocytes are subject to morphological and phenotypic changes, the most thoroughly described being in response to microglial activation by lipopolysaccharide (LPS), inducing reactive astrocytes that produce unknown neurotoxic factors [16,20]. This state is specific to LPS, but it shares many hallmarks of astrocytes in the prion-infected brain [21,22,23]. More generally, reactive astrocytes are often referred to as neurotoxic astrocytes; however, the astrocytic phenotype has been mapped more closely to demonstrate that there are varying states of reactivity associated with disease-specific populations [24]. Although the exact neurotoxic factors they secrete remain unknown, they can also induce a positive feedback loop by producing inflammatory cytokines such as C-C motif chemokine ligand 2 (CCL2) and complement proteins to promote activation and increase the migration of microglia [13,25].

Although they only account for 10–20% of all cells in the brain [13,26], microglia are the first line of defense and arguably have the largest effect on the inflammatory state in the brain (Figure 1). Toll-like receptors (TLRs), namely TLR4, respond to pathogen-associated molecular patterns (PAMPs) such as LPS, and activation of microglia increases TLR4 expression [13,27]. In response, they secrete soluble factors such as cytokines, chemokines, nitric oxide (NO), and reactive oxygen species (ROS) and migrate to the sites of insult. These can act upon astrocytes or directly upon neurons, triggering oxidative stress and even cell death [25,26]. The states of microglia are slightly better defined than those of astrocytes. They generally mimic those of macrophages, and were previously categorized as M1 and M2 activation states. Recently, a movement has emerged to more precisely define microglia activation states to disease-associated microglia (DAM), homeostatic microglia, and subsets thereof [28,29]. This new microglial activation terminology is currently debated and has both pros and cons. For example, various phenotypes of microglia in the terminal prion brain, similar to both M1 and M2, are being considered “DAM” [30].

Regardless of the denotations of DAM and homeostatic, or M1 and M2, these are an oversimplification, and different insults and diseases induce varied phenotypes. Generally speaking, “classical activation” refers to M1 microglia, which are induced by interferon gamma (IFNγ) or LPS and show increased expression of surface markers CD16, CD32, and CD86. M1 microglia upregulate NF-κB-related genes and produce IL1α and IL1β, TNFα, interleukin 6 (IL6), and NO [25,26,29]. These cells undergo morphological changes, with a rounder, amoeboid shape, and decreased process lengths, in conjunction with increased phagocytosis, cytokine release, antigen presentation, and proliferation [31]. This morphology is associated with increased neuronal death, whereas M2 microglia are neuroprotective [32]. M2 microglia were formerly called “resting” microglia, but this term is out of date, as these cells are quite active. This phenotype can be induced by interleukin 4 (IL4) or interleukin 13 (IL13), referred to as “alternative activation.” These cells have increased expression of CD206, and they secrete factors involved in neuroprotection and tissue repair, namely arginase-1 (Arg1) and insulin-like growth factor 1 (Igf1) [26]. These cells are involved in scavenging and the phagocytosis of misfolded proteins and cell debris. They appear ramified, with increased process numbers and lengths for constant surveillance of their environment [31]. A third state, referred to as “acquired deactivation,” is induced by interleukin 10 (IL10) and transforming growth factor beta (TGFβ). It includes microglia with characteristics of both phenotypes as they return from an activation state to a scavenging state [13,26,33]. A microglial cell will change activation states multiple times throughout its life, as they can live to be more than four years old. Their activation states also influence neighboring astrocytes. As M1 microglia secrete factors that shift astrocytes toward a reactive phenotype, M2 microglia produce anti-inflammatories such as IL10 and TGFβ that shift astrocytes toward a homeostatic phenotype [13].

Importantly, the molecular milieu produced by M1 microglia negatively regulates that of M2 microglia and vice versa, supporting a tightly regulated process, potential for phenotype switching, and resulting in an environment in the brain that favors one state of activation over another [26,32].

## 3. A Constellation of Astrocytes

Understanding inflammation and the contributing roles of astrocytes and microglia is critical for early diagnostics and the development of robust therapeutics for prion disease. Microgliosis and astrogliosis, with concomitant genetic and proteomic changes, occur in both humans and animal models and is present before the onset of clinical signs [21,22,23,30,34].

S100 calcium-binding protein β (S100β), a general astrocyte marker, has been cited as a reliable biomarker of CJD and scrapie when detected in the cerebrospinal fluid and serum of humans and animals, respectively [35,36,37]. S100β, in combination with the complement protein C3, is a marker for reactive astrocytes in prion and other neurodegenerative diseases [16], as is lipocalin 2 (LCN2) in combination with C3 [38]. LCN2 is a protein secreted by astrocytes in inflammatory conditions that contributes to apoptosis and morphological changes [39]. Guanidine-binding protein 2 (GBP2) is another marker of neurotoxic astrocytes that is highly abundant in the brains of prion-infected animals and CJD patients [16,22]. Although still poorly understood, these reactive astrocytes are known to secrete toxic mediators, such as saturated lipids, that contribute to neuronal cell death [16,20,22,40,41]. The degree of astrocyte reactivity induced by prion infection is inversely correlated to the incubation time, as demonstrated in multiple strains of mouse-adapted scrapie, with disease occurring more rapidly in the strains with increased astrogliosis [42].

Recently, astrocyte-derived saturated lipids from apolipoprotein E (APOE) and apolipoprotein J (APOJ) have been shown to induce neuronal cell death in vitro and in an in vivo model of acute axonal injury [40]. Interestingly, APOE has been shown to be upregulated in prion mouse models [23], particularly in microglia [43]. However, knockout (KO) of this protein was not protective and resulted in decreased incubation time, increased inflammatory cytokines, increased markers of reactive astrocytes, increased markers of both DAM and homeostatic microglia, and neuronal loss. Additionally, microglia in APOE KO mice show an impaired ability to clear PrP^Sc^ and damaged neurons [43]. Although this does not completely exclude the potential involvement of astrocyte-derived saturated lipids in prion pathogenesis, it does suggest that there may be other neurotoxic factors, as knockout of APOE would be expected to restore health in infected animals.

Both astrocytes and neurons can be infected with PrP^Sc^ [44], and astrocytes demonstrate the ability to transfer PrP^Sc^ between each other and to neurons in primary cell culture [45]. Astrocytes more readily take up PrP^Sc^ than neurons. It is unknown whether this is due to an increased susceptibility or whether it a mechanism to protect the neurons. Multiple studies have interrogated how PrP expression by different cell types affects disease susceptibility. Seminal studies used transgenic mice lacking murine PrP but expressing hamster PrP in only astrocytes or neurons via the GFAP or enolase promoter, respectively. They were found both to be susceptible to infection with hamster prions, although with longer incubation periods than infected wild-type hamsters. Mice that expressed astrocyte-specific hamster PrP had longer incubation periods than those with neuron-specific hamster PrP [46,47,48]. Interestingly, the survival times were similar between transgenic mice expressing neuronal hamster PrP and those that ubiquitously expressed hamster PrP, depending on route of inoculation. Neurodegeneration was abundant in all the transgenic mice, but retinal degeneration was limited to mice expressing PrP in both neurons and astrocytes [48]. A more recent study contradicts these earlier studies by suggesting that only neuronal expression of PrP is required for the disease to be fatal. The *Cre-Lox* system was used to express PrP under the Synapsin1 promoter or GFAP promotor for neuron- or astrocyte-specific PrP expression, respectively. When PrP was expressed solely in astrocytes, there was significant PrP^Sc^ accumulation but no microgliosis, astrogliosis, vacuolization, or neuronal loss, and mice do not develop signs of clinical disease. When PrP was restricted to neurons, mice developed all the classic signs of prion disease but had less gliosis and lived significantly longer than wild-type animals. This suggests that astrocytes become infected and that further disseminating prions can accelerate disease and promote inflammation, but that they are not required for the disease to occur [44]. Although the findings of these studies may not align, together, they suggest that PrP expression in neurons or astrocytes may have different impacts on disease incubation time and pathogenesis depending on the prion strain, route of inoculation, and tissue type.

Microglia follow a similar pattern of dispersal throughout the brain to PrP^Sc^ [49,50], likely due to their role in phagocytosis. Astrocytes, however, show a much different pattern of activation that is not consistent with PrP^Sc^ deposition, and different regions of the brain display different astrocyte morphologies. This is particularly apparent in the hippocampus and thalamus. There are multiple strains of mouse-adapted scrapie that have been developed for research purposes, including 22L, RML, and ME7, which show different PrP^Sc^ depositions but similar gliosis [21]. Intriguingly, in 22L-infected mice, more microglia are observed in the thalamus compared to the hippocampus, but more astrocytes are observed in the hippocampus compared to the thalamus [50]. Similar observations have been made for RML [51]. This suggests that astrocytes and microglia are working somewhat independently of one another. Note that these observations are based solely on the GFAP and Iba1 expression of astrocytes and microglia, respectively, and does not delve into the molecular profiles of these cells.

A recent study in hamsters in vivo and a primary cell model found varying susceptibility of glia to prion propagation, dependent on both the brain region and the prion strain. Moreover, glia exposed to lipopolysaccharides to elicit an immune response prior to infection did not replicate prions as effectively as glia that were exposed to dexamethasone, an inhibitor of innate immunity. These findings suggest that the inflammatory status of glial cells may contribute to their ability to replicate and disseminate prions [52].

A healthy mouse contains seven distinct types of astrocytes with different levels of protein expression and distribution throughout the brain based on single-cell profiling [53]. The profiles of astrocytes in response to prion disease have only begun to be interrogated, but neurotoxic astrocytes appear to have the same genetic signature regardless of prion strain [42]. Although more phenotypes have been uncovered, the binary nomenclature of A1 and A2 astrocytes are most widely utilized, as they are currently the best-characterized phenotype. Distinctly, there are more phenotypes and disease-associated astrocytes which require greater characterization and not simply binary division of reactive astrocytes as ‘good vs. bad’ [24]. Interestingly, translational profiling of cells in the prion-infected brain shows an increase in both A1 (pro-inflammatory) and A2 (anti-inflammatory) astrocytes as the disease progresses, despite A2 being generally associated with homeostatic function and the maintenance of neuronal health [54]. Indeed, a recent single-cell analysis of prion-infected mouse hippocampus and cortex did not identify large populations of reactive astrocytes but instead identified populations of dysfunctional homeostatic astrocytes [30]. Together, these findings suggest that a subset of astrocytes may be contributing to preventing neurodegeneration, even in the late stages of the disease. There is evidence that this process is tightly regulated by crosstalk between astrocytes and microglia, as prion mouse models with microglia ablation show drastically different astrocyte profiles, including increased expression of genes associated with reactive, pro-inflammatory astrocytes [23].

Transitioning from a “resting” astrocyte to a neurotoxic astrocyte may require microglia-derived C1qa, IL1α, and TNFα [16]. Logically, abolishment of these signals, here referred to as triple knock-out, should prevent neurodegeneration or at least decrease neuroinflammation in the context of prion disease. However, removal of these inflammatory proteins, although successful in abolishing C3+ astrocytes, also accelerated disease in mouse models [22]. No changes were detected in vacuolization, PrP^Sc^, GFAP (a marker of astrocytes) or ionized calcium-binding adaptor molecule 1 (Iba1) (a marker of microglia). However, homeostatic microglia, marked by the expression of transmembrane protein 119 (TMEM119) and purinergic receptor P2Y12 (P2RY12) [34,55], were significantly decreased in the prion-infected triple knock-out mice [22]. This phenomenon appears to be unique to prion disease, as inhibition of C1qa and C3 has shown to be neuroprotective in animal models of other neurodegenerative diseases [16,56,57]. This suggests that, as microglia are critical for astrocyte activation, reactive astrocytes may indeed be critical in regulating microglia phenotypes as well, and that this process is important for combatting prion infection. This protective role for microglial C1qa and TNFα in prion disease in the CNS contrasts with the facilitative role observed for C1qa, C3, and TNFα in peripheral prion pathogenesis [58,59,60].

Astrocytes contribute to the proper maintenance of the blood–brain barrier (BBB), which is known to break down in prion disease. The mechanism behind this is still poorly understood, but a recent study by Kushwaha and colleagues found that reactive astrocytes from prion-infected mice secrete factors such as IL-6 that contribute to a disease-associated phenotype in endothelial cells. These cells showed dysregulation of tight and adherence junctions and trans-endothelial electrical resistance, which are critical for the maintenance of a proper BBB, when co-cultured with astrocytes or exposed to astrocyte-conditioned media from infected mice. Intriguingly, this phenotype was partially reversed when the endothelial cells were exposed to extracellular vesicles from healthy astrocytes [61]. Astrocytes are critical for neuronal health and BBB integrity, but in the context of prion and other neurodegenerative diseases, astrocytes become reactive, change morphology, and secrete inflammatory molecules and other unknown factors, which can wreak havoc in the brain.

## 4. DAM! Look at All Those Microglia

The role of microglia in prion disease remains controversial, as studies show that they are critical, expendable, and everything in between. Ultimately, this depends on the mechanism used to decrease or eliminate microglia, as well as the time point at which this is done.

The microglial contribution to neuronal toxicity in prion disease was first described in 1998 by Giese et al. They co-cultured primary microglia and neurons infected with PrP^Sc^ and ascertained that having microglia present in the culture was critical for PrP^Sc^-induced neurotoxicity. This finding was corroborated using three different scrapie mouse models, for each of which PrP^Sc^ accumulation correlated strongly with microglial activation, which preceded neuronal cell death [62]. Compared to the other cell types in the brain, microglia express much less PrP [63].

Conversely, other studies demonstrate that microglial depletion exacerbates prion pathology, suggesting that microglia are protective in prion disease [64,65,66,67]. Microglia appear to be effective in phagocytosis and the clearance of apoptotic neurons and cellular debris but inefficient in phagocytosing PrP^Sc^ [68]. Thus, the mechanism behind this putative microglial protection remains unclear.

The proteins involved in the complement cascade, namely C1q, C3, C4, and the C3 receptor on microglia, have been implicated in phagocytosis and over-pruning of synapses in mouse models of Alzheimer’s and other diseases [56,69]. Inhibition of these proteins leads to improved neuronal health. However, efficient phagocytosis of PrP^Sc^ by microglia has yet to be consistently shown for prion disease.

Misfolded PrP, including PrP^Sc^ and PrP_106-126_, a peptide that is known to mimic the neurotoxic effects of PrP^Sc^ [70], induces the production of nitrite, inducible nitric oxide synthase (iNOS), ROS, IL1β, and IL-6. Microglial-derived IL-6 has been shown to induce neuronal cell death [71,72,73]. PrP^Sc^ exposure in BV2 microglial cells and primary microglial cultures demonstrate similar inflammatory, neurotoxic-associated profiles [72,74,75]. Phagocytosis of PrP_106-126_ by both BV2 and primary microglia increases mRNA for TNFα and iNOS, markers of classically activated or M1 microglia, and decreases mRNA for Arg1 and mannose-receptor c-type 1 (Mrc1), markers of alternatively activated or M2 microglia. It also appears to have little effect on mRNA for suppressor of cytokine signaling 3 (SOCS3) and interleukin 4 receptor alpha (IL4Rα), markers of acquired deactivation [33,73,76].

A single-cell analysis showed that microglia in the prion-infected brain may indeed by classified into multiple subtypes: homeostatic microglia that express *TMEM119*, *P2RY12*, and *CXC3R1*, and four variations of activated cells: proliferating, phagocytic, type I interferon-responding, and antigen-presenting [30]. Ribosomal and transcriptomics studies have found that changes in microglial occur much earlier in disease, whereas changes in neurons do not occur until late clinical stages [54,77,78]. In fact, a study by Vincenti and colleagues showed that the most profound gene changes throughout the course of prion infection in mice were in the microglia. They found microglia to have a highly proinflammatory signature, in addition to an upregulation in genes involved in metabolism and protein translation and processing [79]. Carroll et al. made similar observations when analyzing gene expression throughout the time course of RML-scrapie in mice. There was an increase in the genes involved in proinflammatory microglia and proliferation at the preclinical and clinical stages of the disease, and more changes were seen in microglial genes than in any other cell type. Intriguingly, they did not see gene expression in microglial genes that was consistent with the neurotoxic microglia that were previously reported for other neurodegenerative diseases [23,80].

TMEM119 and P2RY12 expression is associated with ramified microglia, containing small cell bodies and long, thin processes [55]. P2RY12 is highly expressed on ramified processes and is the first receptor stimulated in the process of microglial activation, causing processes to extend to sites of injury. P2RY12 expression decreases as microglia migrate and undergo a shift to an amoeboid state, increasing phagocytic and secretory activities [81]. In some neurodegenerative diseases, P2RY12 can be associated with activated, inflammatory microglia [81,82], but in the context of prion disease, both P2RY12 and TMEM119 are shown to decrease over the course of infection [30,34]. Changes in microglia are first detectable in the thalamus at preclinical stages, and cell morphology shifts from being ramified to activated or amoeboid. This is particularly apparent in the loss of P2RY12+ and TMEM119+ microglia in the thalamus [34], while Iba1+ microglia increase in number and appear more amoeboid with thick processes in both the thalamus and hippocampus [34,83]. A recent study additionally established the ligand Galectin 3 (Gal-3) and its receptor TREM2, which are involved in microglia activation and M1 polarization, to colocalize with Iba1+ microglia in terminal mice with different strains of prion disease [84].

PrP^Sc^ activates microglia and polarizes them toward a classically activated phenotype [72]. However, the anti-inflammatory cytokines TGFβ, IL10, and IL13 are upregulated in the brain at late stages of prion disease [85], as are markers of alternative activation and acquired deactivation [54,86]. Because these activation types are not being induced by microglia upon prion infection, this suggests that crosstalk between cells is responsible for producing factors that polarize microglia from a DAM state to a homeostatic phenotype, likely in an attempt to attenuate inflammation. In fact, studies have shown that knockout of IL10 in mice lead to increased susceptibility to disease [85], suggesting that cytokines, particularly those responsible for acquired deactivation, are critical in dampening prion-induced inflammation. Similarly, ablation of CD14, a GPI-anchored protein that interacts with toll-like receptors, led to an increase in activated microglia in prion-infected mice. These mice had increased expression of IL10 and TGFβ and survived longer than wild-type infected mice [87].

Are microglia protective to neurons? Or are they contributing to neurodegeneration? To help answer this question, multiple studies have investigated the effects of decreasing or removing microglia on the course of prion infection.

Microglial proliferation and survival require continual stimulation by the tyrosine kinase macrophage colony stimulating factor receptor (CSF1R) [88]. CSF1R is activated by colony stimulating factor 1 (CSF1) and interleukin 34 (IL34), which are produced by astrocytes and neurons. CSFR1 stimulation induces the expression of PU.1 and C/EBPα, which drive the renewal, differentiation, and survival of microglia [89]. Both PU.1 and C/EBPα are increased in the brains of CJD patients. The function of CSF1R can be inhibited by the drug GW2580 [83]. Infected mice that received this drug showed a decrease, but not ablation, in microglia, a decrease in PU.1 and C/EBPα, and a modest increase in the markers of M2 microglia. Moreover, these mice had improved neuronal health and increased survival compared to vehicle-treated mice [83]. A second study used CSF1R signaling to modify microglial numbers. Mice with prion infection were either subjected to an increase in microglia proliferation via stereotaxic injection of CSF1 or IL34 to increase the function of CSF1R, or to GW2580 to inhibit the receptor and decrease microglial numbers [90]. Conversely to the previous study, increases in microglia were associated with improved neurogenesis and neuronal development in the hippocampus. Microglial-derived TGFβ was determined to be responsible for this, as neurogenesis was decreased when TGFβ was inhibited [90]. Another study knocked out IL34 in mice that overexpressed PrP, which led to a decrease in microglial numbers. They observed shorter incubation times with prion infection and an increase in PrP^Sc^. They saw similar effects when they ablated microglia from mouse ex vivo organoid cultures, in addition to more neuronal loss, decreased TNFα and IL1β mRNA, and increased CCL2 and CCL5 mRNA [65].

Another study used PLX5622, another inhibitor of CSF1R, to decrease the number of cortical microglia by as much as 90%. This was associated with decreased survival when the drug was administered at 14 days post infection (dpi) or at the preclinical stages of the disease (80 dpi). Although the microglia were decreased, the mice showed increased vacuolization, astrogliosis, and, importantly, similarly distributed but significantly increased PrP^Sc^, suggesting an important role for microglia in the clearance of prion aggregates. These results were consistent amongst three different mouse-adapted scrapie models [66]. Similar to the previous study, key inflammatory cytokines such as TNFα and IL1α were decreased in PLX5622-treated mice [66], suggesting that microglia may be a significant source of these inflammatory mediators and suggesting that elimination of inflammation may not be completely protective.

Generally, these studies demonstrate a protective role for microglia in prion disease and suggest that they play a part in PrP^Sc^ clearance. However, a recent study does not support this role. Bradford et al. targeted CSF1R by deleting an enhancer of the receptor that prevents microglial development but allows mice to develop normally. These mice are referred to as *Csf1r*^ΔFIRE^ and have complete microglial knock-out [64]. *Csf1r*^ΔFIRE^ mice succumbed to the disease more rapidly, solidifying a role for microglia in neuroprotection. Similar to the previous experiments, TNFα mRNA was significantly decreased, further identifying this as a microglia-derived cytokine. The brains of *Csf1r*^ΔFIRE^ mice showed significantly more vacuoles, but the neuronal counts in the CA1 region of the hippocampus were unchanged. No differences were seen in PrP^Sc^ using immunohistochemical staining, Western blotting, or seeding activity of PrP^Sc^ [64]. These animals showed decreased GFAP+ astrocytes in the hippocampus compared to wild-type controls, although the total brain mRNA for GFAP was the same. Additionally, the mRNA for GBP2, a gene associated with reactive astrogliosis [22], was decreased [64]. Interestingly, an increase in reactive astrocytes was observed in the superior colliculus, as more C3+/LCN2+/GFAP+ astrocytes were detected using immunofluorescence. GFAP+ astrocytes appeared to have enhanced phagocytosis in the absence of microglia, as these cells showed increased uptake of neuronal synapses. Astrocytes also showed increased unfolded protein response (UPR) early in the disease, associated with an increase in phosphorylated PERK and eIF2α [64]. The overall findings of this study corroborate the role of microglia in neuroprotection against prions and also highlight their regulatory role in astrogliosis, as astrocytes appear to compensate for microglia by increasing phagocytosis and UPR functions, with deleterious effects [38,64].

A recent study showed the effects of intermittent treatment of PLX5622 (an inhibitor of CSFR1 used in a previously mentioned study) on microglial proliferation throughout prion disease to identify when these cells are most beneficial. Oral administration of this drug to mice can decrease microglia by inducing caspase-3-mediated apoptosis, resulting in an up to 90% decrease in microglial numbers in 7 days. Race et al. ablated microglia 7 days prior to infection with prions. Treatments were continued to 7 dpi, 77 dpi, or 112 dpi (mean survival is ~150 dpi), resulting in a significant reduction in Iba1+ microglia but no changes in PrP^Sc^ seeding activity. Early treatment with PLX5622 (prior to inoculation) did not change susceptibility to prion infection [88]. It was established that microglial morphology and function changes with age, characterized by increased inflammatory mediator production and decreased phagocytic abilities [28]. Consistent administration of PLX5622 is necessary to maintain decreased CNS microglia, and cells return rapidly after dosing ends. These new microglia are similar to those present in young mice. Therefore, Race et al. hypothesized that reintroduction of healthy microglia may be beneficial in attenuating prion disease. Beginning at 80 dpi, mice were treated for one week with PLX5622 either once, two times with two weeks between treatments, or three times with two weeks between treatments. Regardless of when or how frequently the drug was administered, the mice succumbed to the disease at the same time, and no significant changes in Iba1+ cells were observed. Uninfected mice, however, showed a decrease in Iba1+ cells with treatment, suggesting that the drug was working but was ineffective in reducing microglia at late stages of the disease [88]. Finally, to further interrogate the role of microglia in potentially decreasing PrP^Sc^ accumulation, they utilized another model of mice, called tga20 mice, that express PrP at 6- to 10-fold normal expression and succumb to the disease more rapidly than WT mice but have less than 10% the amount of PrP^Sc^. These mice were treated with PLX5622 beginning at 14 dpi, and for the duration of the study, they showed significant reductions in Iba1+ microglia. Tga20 mice treated with PLX5622 showed a slight but significant decrease in survival time compared to untreated tga20 mice [88], whereas this survival time difference was much greater in this group’s previous study using WT mice that showed significant PrP^Sc^ accumulation [66]. Together, these data suggests that microglia do not play a significant role in prion pathogenesis early in the disease, but they show benefits at later time points in the disease, and one of these benefits may be the clearance of PrP^Sc^ itself.

Another recent study looked at the phagocytic activity of both microglia and astrocytes from mice at terminal stages of prion disease. In purified cultures of glia isolated from terminal mice, astrocytes showed a decreased ability to phagocytose, while microglia showed an increased ability, including the upregulation of many related genes. Microglia showed an increase in phagocytic activity, including engulfment of neurons in various brain regions at terminal stages [76]. This study did not look into whether or not these neurons were infected with PrP^Sc^, as microglia may be phagocytosing neurons to prevent further prion propagation.

Altogether, what do these experiments tell us? Having a balance of microglia is important, and this can be hard to achieve. Whether or not microglia improve survival may be dependent on the drug or transgenic model used. However, overall, it appears that microglia are beneficial to the host in prion infection. Although classically activated phenotypes may be responsible for the production of inflammatory cytokines that may promote astrogliosis and neurotoxicity [83,90], the alternatively activated and acquired deactivated phenotypes appear to be neuroprotective and mediate astrocyte reactivity [90]. Phagocytosis of PrP^Sc^ may be important later in the course of the disease, but this may also lead to the phagocytosis of neurons [66,76,88]. Moreover, in the absence of microglia, astrocytes compensate by increasing dysregulated phagocytosis and UPR activities, which contribute to neurodegeneration [38,64].

## 5. Conclusions

Studying the role of glial cells in prion disease has its limitations in animal models. Microglia are critical for brain development, and animals cannot survive without astrocytes, so animal models require the use of drugs to decrease cell numbers or cell-specific mutations. This greatly increases the number of variables that can change when designing an experiment, such as at what point to deliver drugs or if mutations may have off-target effects. Primary cell culture is generally limited to one or two cell types and therefore may not be representative of what is occurring in the brain. Moreover, the neurotoxic effects of prions are not always able to be fully recapitulated outside of the brain [91]. Little more can be learned from human patients, as glial cells can only be studied in post-mortem tissues, allowing only inference of the role of these cells in terminal disease stages. Although they do not fully recapitulate cells in the aged brain, various cell types, including astrocytes, neurons, and microglia, can be derived from mouse neural stem cells and human induced pluripotent stem cells (iPSCs) [92]. Moreover, iPSCs can be used to develop cerebral organoids, which may provide valuable insight into the neural networks and interactions between cell types in the human brain [93,94]. Animal models and the technologies to research them continue to improve. For example, live cell imaging of glia in the brains of prion-infected mice may give valuable insights [95]. Mouse models continue to improve as well. For example, *Cre* recombinase mice have been developed that are more selective for astrocytes, such as *Aldhl1l*, as opposed to *GFAP*, which is also expressed in neurons and may lead to off-target effects [96].

Despite these caveats, there have been many elegant studies over the last decade that have expanded our knowledge of the role of astrocytes and microglia in prion disease. In general, these studies have shown that although astrocytes are critical for maintaining neuronal homeostasis in a healthy brain, they can become reactive and neurotoxic in the prion-infected brain, contributing to a weakening of the blood–brain barrier and the production of neurotoxic factors [41,61]. Further studies are necessary to determine what these neurotoxic factors are in the context of prion disease. However, removal of the signaling molecules required for the development of classic “reactive” astrocytes actually decreased the survival time of prion-infected animals, suggesting that the presence of these cells may have some benefit to the host [22]. There is further evidence that not all of the astrocytes in a prion-infected brain contribute to neuronal death, as phenotypes of homeostatic “A2” astrocytes persist, even at terminal stages of the disease [23,54].

Crosstalk between astrocytes and microglia appears to have both benefits and drawbacks. In the absence of microglia, astrocytes increase phagocytosis of neuronal synapses, further contributing to neuronal loss [64]. Activated microglia secrete a milieu of inflammatory cytokines and molecules that induce a reactive phenotype in astrocytes. Those that are shown to be critical are TNF, C1qa, and IL1α, but this is in response to LPS, and microglia may secrete different molecules in response to infectious prions [16,22]. In turn, astrocytes can signal to microglia with molecules such as CCL2 and complement proteins, contributing to morphological and phenotypic changes in microglia towards a disease-associated state [13,25].

The role of microglia in prion infection may be slightly more controversial than that of astrocytes. The role of microglia in PrP^Sc^ dissemination and clearance remains unclear [62,68,71,74,97,98]. Single-cell and whole-brain gene analyses suggest that there are multiple populations of microglia in the prion-infected brain, but they may not directly contribute to neurodegeneration [23,30,54,79]. Microglia with a neurotoxic phenotype that have been described in other neurodegenerative diseases have yet to be described for prion disease, although these cells may still be indirectly contributing to neuronal death [16,26,29,56,80]. Microglia in the prion-infected brain generally appear more amoeboid, with a decrease in process length and markers of homeostasis, although homeostatic microglia still persist even at the clinical stages of the disease [23,34,54,83]. Decreasing or altogether eliminating microglia in the prion-infected brain generally leads to acceleration of the disease, although there is contradictory evidence in their role in phagocytosing and removing PrP^Sc^ [64,65,66,76,88]. Overall, the current literature suggests that microglia and astrocytes tightly regulate one another and maintain a balance in protecting neurons and contributing to the clearance of infected neurons.

Future studies should focus on understanding the specific pathways involved in gliosis in the prion-infected brain. Many questions remain unanswered, such as what neurotoxic factors are produced by reactive astrocytes and what initiates a breakdown of their blood–brain barrier maintenance. Although the general consensus on microglia is that they play a neuroprotective role, it remains unclear what factors they secrete or how their migratory and phagocytic functions may be compromised in response to prion infection. Therapeutic intervention should focus on bolstering the neuroprotective functions of both astrocytes and microglia while inhibiting their neurotoxic functions.
